# Ascending aortic aneurysm associated with tetralogy of Fallot in an adult mare

**DOI:** 10.1002/vms3.311

**Published:** 2020-06-23

**Authors:** Valentina Vitale, Gaby Van Galen, Malene Laurberg, Bridgette Young, Victoria Mciver, Marta Wereszka, Marina Gimeno

**Affiliations:** ^1^ University Teaching Hospital Sydney School of Veterinary Science University of Sydney Camden New South Wales Australia

**Keywords:** aortic aneurysm, cardiac failure, congenital disease, Equine

## Abstract

Congenital heart disease is relatively uncommon in horses. Only three reports exist that describe a tetralogy of Fallot in adult horses. Whereas in humans the presence of congenital heart disease constitutes a risk factor for developing aortic aneurysms, their association has never been reported in horses. Here, we present a case with a large ascending aortic aneurysm as a complication of a tetralogy of Fallot in an adult mare. The mare was referred with sustained tachycardia and a 5 days history of mild intermittent colic, depression, inappetence and weight loss. Echocardiography was used to characterize the cardiac abnormalities and aortic root dilation. Due to the poor prognosis, the mare was euthanized and *post‐mortem* examination further characterized the abnormalities. At least four factors contributed in this case to the development of aortic haematoma: congenital disease, mucoid extracellular matrix accumulation *vasa vasorum* dysfunction and inflammatory/degenerative lesions in the aorta's intima. Although colic is primarily caused by gastrointestinal issues, cardiac disease should be suspected in cases with sustained tachycardia, even in absence of murmurs or arrhythmias. Despite the fact that congenital abnormalities are usually detected in foals, they may sometimes remain unnoticed for several years.

## Graphical abstract

Here, we present a case with a large ascending aortic aneurysm as a complication of a tetralogy of Fallot in an adult mare. She was referred with sustained tachycardia and a 5 days history of mild colic pain. Echocardiography allowed to identify the congenital disease and the aortic root dilation. Due to the poor prognosis, the mare was euthanized and the post‐mortem finding allowed closer characterization of the abnormalities.

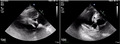

## 1 INTRODUCTION

Congenital heart disease (CHD) represents the outcome of errors during fetal cardiac development (Scansen, 2019). The reported prevalence of 0.1%–0.5% for congenital cardiac defects in horses is relatively low as compared with other animals (Hall, Magdesian, & Kitt, 2010; Kruger, Wunschmann, Ward, & Stauthammer, 2016). Such defects have usually a profound impact on the foals through altered growth rate, exercise intolerance, respiratory distress and death (Hall et al., 2010; Scansen, 2019). Tetralogy of Fallot (TOF), a cardiac anomaly consisting of an overriding aorta, a ventricular septal defect, pulmonic stenosis and right ventricular hypertrophy (Cargile, Lombard, Wilson, & Buergelt, 1991; Gesell & Brandes, 2006), has a reported incidence of 28% of all forms of CHDs (Hall et al., 2010). The condition has been reported in a large variety of breeds (Cargile et al., 1991; Scansen, 2019) but is most often described in neonatal foals (Gesell & Brandes, 2006; Hall et al., 2010). Only three reports exist from adult horses (Cargile et al., 1991; Gesell & Brandes, 2006; Taulescu et al., 2016).

Whereas in humans the presence of CHD constitutes a risk factor for the development of aortic aneurysms (Billaud, Hill, Richards, Gleason, & Phillippi, 2018; Mork, Eftekhari, Tang, & Klaaborg, 2019), there are no reports of their association in horses. Aortic aneurysms are rare in horses and occur mainly near the aortic arch. Aortic aneurysms have been reported in horses as an acquired condition, a congenital anomaly or associated with chronic aortic regurgitation or Strongylus vulgaris. In most cases aneurysms were detected at *post‐mortem* examination after aortic rupture (Decloedt, 2019). The rupture of the aneurysm usually results in sudden cardiac death (SCD) but less acute signs of colic, anorexia, recumbency, depression, poor performance, coughing and peripheral oedema have also been reported (Decloedt, 2019; Marr et al., 1998; Ploeg et al., 2013). The majority of aortic ruptures have been documented in Friesian horses (Ploeg et al., 2013), and thought to be related to primary changes in the vascular connective tissues (Ploeg et al., 2015; Tursi, Poser, Janus, & Guglielmini, 2018).

The present communication describes for the first time in a horse a large ascending aortic aneurysm as a complication associated with a Tetralogy of Fallot.

## 2 CASE REPORT

### 2.1 Case history

A 5‐year‐old Australian Stock Horse mare was referred to the – blocked for peer review – with a sustained tachycardia of 80 bpm during the last 12 hr and a 5 days history of mild intermittent colic, depression, inappetence and weight loss. The mare had no previous history of illness and vaccinations and deworming treatments were up to date. The mare was not exercised on a daily basis, but had participated in low‐level reining competitions over the past year. There was no history of poor performance or exercise intolerance.

### 2.2 Clinical findings

On admission the mare was bright, alert and responsive with no evident signs of pain. Her body weight, height at withers and body condition score (BCS) on arrival were 563 kg, 150 cm and 6/9 (Henneke, Potter, Kreider, & Yeates, 1983), respectively. Mucous membranes were pink and moist but capillary refill time was prolonged and extremities were cold. The mare had tachycardia (80 bpm) with a respiratory rate of 20 rpm and normal rectal temperature (38.3°C). Peripheral pulse was normal and all palpable lymph nodes were within normal limits. The rectal exam and abdominal ultrasound were unremarkable. Peritoneal fluid was macroscopically normal and had a low lactate concentration. Initial complete blood count and serum biochemistry revealed a normal packed cell volume (34%) and blood lactate (1.3 mmol/L), but moderate leukocytosis (16.63 × 10^9^/L; reference range, 4.9–11.10 × 10^9^/L) with neutrophilia (11.59 × 10^9^/L; reference range, 2.5–6.9 × 10^9^/L). There was also hyperproteinemia (92 g/L; reference range, 56–79 g/L), hyperfibrinogenemia (7 g/L; reference range, 2–4 g/L) and increased serum amyloid A (999 µg/ml; reference range, <10 µg/ml). Cardiac troponin I concentration was 89 ng/L (reference range, <100 ng/L). As no evident cause of gastrointestinal pain was identified, a cardiac cause of tachycardia was suspected. A Holter monitor (Televet® 100 ECG & Holter®, Jørgen Kruuse, Denmark) showed sinus tachycardia (80–100 bpm) throughout the night. A brief ultrasound confirmed there was no pericardial effusion and a complete echocardiography was scheduled for the following day. Meanwhile, the mare was hospitalized and treated with IV fluid therapy (Hartmann's solution) and flunixin meglumine (1.1 mg/kg IV q12hr, Flunixil ilium®, Troy laboratories Pty limited). Based on the history the mare had been inappetent during the last few days, so gastric ulceration was considered likely to be present and a possible cause of pain thus ranitidine (6.6 mg/kg PO q8hr, Ulcerguard®, Ranvet Pty Ltd) was added to the treatment. During the night the mare presented several episodes of uncomfortable behaviour expressed with stretching, strange postures with hindlimbs and restlessness that responded favourably to the administration of butorphanol (0.03 mg/kg IM, Torbugesic®, Zoetis Australia Pty Ltd). A small amount of normal faeces was produced overnight.

The following morning the mare developed acute severe pain with pawing, sweating and repeated attempts to lie down. She had recently received one scheduled dose of flunixin meglumine, thus repeated doses of detomidine (8 µg/kg IV, Sedator®, Randlab Australia Pty Ltd) and butorphanol (0.03 mg/kg IV, Torbugesic®, Zoetis Australia Pty Ltd) were administered but she remained severely painful. Rectal palpation and abdominal ultrasound were unchanged but due to her continuous attempts to lay down it was considered unsafe to repeat the abdominocentesis. Blood lactate increased to 2.9 mmol/L (reference range, <2 mmol/L). Due to concerns of concomitant new or undetected severe gastrointestinal condition, a decision to perform an exploratory celiotomy was taken.

### 2.3 Surgical findings

Following premedication (22,000 IU/kg IM procaine penicillin, Propercillin ilium®, Tory laboratories Pty limited, and 6.6 mg/kg IV gentamicin, Gentam 100 ilium®, Troy laboratories Pty limited) the mare was induced with glyceryl guaiacolate ether (51 mg/kg IV) and ketamine (2.2 mg/kg IV, Ketamil ilium, Troy laboratories Pty limited) and maintained in dorsal recumbency with isofluorane in 100% oxygen. Exploratory laparotomy with ventral midline incision revealed a right dorsal displacement with 180° large colon torsion with no apparent vascular compromise. Intestinal contents were evacuated via a pelvic flexure enterotomy and a biopsy sample of large colon was obtained from the pelvic flexure. The surgeons suspected that the heart was abnormally enlarged as it could be felt readily when palpating the diaphragm.

During anaesthesia blood pressure was maintained within 60–80 mmHg with dobutamine infusion (0.2–4 µg kg^−1^ min^−1^) and she remained tachycardic (70–80 bpm). Blood gas analyses, performed at 30 and 80 min after induction, showed mild hypercapnia (48.7 mmHg) and mild lactic acidosis (lactate = 3.3 mmol/L).

Immediately after recovery, the mare developed laryngeal collapse and an emergency tracheostomy restored the airway. The mare remained uncomfortable with a sustained tachycardia of 80–100 bpm. Additional analgesia with morphine (0.1 mg/kg, IM) was provided and 3 hr after recovery echocardiography was performed.

### 2.4 Echocardiography

**FIGURE 1:**
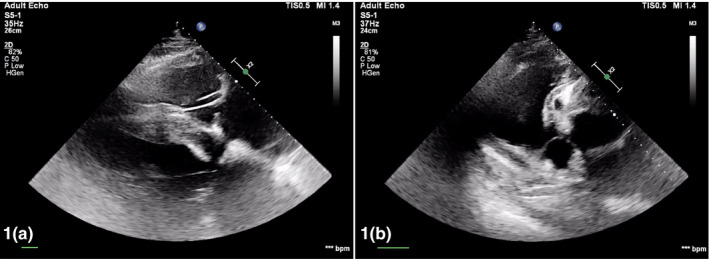
(a) Right parasternal long‐axis four‐chamber view: marked right ventricle and atrium dilation; (b) Right parasternal view right ventricle outflow: aorta and pulmonary artery seem to join together to communicate with the right ventricle

A transthoracic echocardiographic examination was carried out according to a previously published protocol (Marr & Patteson, 2010) using an ultrasound system (Philips EPQ 5G, Release 1.5.2) with a 2.5 MHz phased‐array transducer with harmonic imaging. At the right parasternal long‐axis four‐chamber view, severe dilation of right ventricle and atrium and increased thickness of interventricular septum were evident (Figure 1a). The aorta appeared dilated compared with the cardiac chambers (aortic diameter of 7 cm), thick walled and overriding. A doubly committed ventricular septal defect (VSD) of 3.67 cm was identified just below the aortic valve. The shunt between right and left heart was principally left‐to‐right but during some contractions right‐to‐left shunt was visible with colour Doppler during systole. The pulmonary artery, close to its valves, was in continuity with the aorta and in communication with the VSD (Figure 1b). The diameter of pulmonary artery was markedly reduced (3.94 cm). A communication of 1.27 cm between the aorta and right atrium with a left‐to‐right shunt was also identified with colour Doppler. At the base of the heart a large cavity with an internal diameter of approximately 15 cm was observed in communication with the aortic root. At this level, the aortic wall appeared markedly thickened (1.54 cm) and the thickness of the walls of the cavity was approximately 1 cm. Due to the cardiomegaly, the altered anatomy and noncompliance of the animal, obtaining standard imaging views was challenging. Recorded standard measurements are reported in Table 1.

**TABLE 1:** The measured cardiac dimensions in our mare are compared with reference ranges (Marr & Patteson, 2010)

Parameter	Patient	Normal ranges (ponies)	Normal ranges (horses)
Aorta diameter (cm) (Right parasternal short axis view)	7.00	5.0 ± 1.0	7.8 ± 0.6
PA diameter (cm) (Right outflow view)	3.94		5.41 ± 0.38
RVIDs (cm)	4.93		
RVIDd (cm)	6.47		
IVSs (cm)	5.86	3.8 ± 0.5	4.6 ± 0.5
IVSd (cm)	5.02	2.4 ± 0.2	2.8 ± 0.2
RA diameter (cm)	9.29		
LA diameter (cm)	10.96		11.4 ± 0.5
Aorta/PA ratio	1.7		1.0–1.5
LA/Aorta ratio	1.5		1.6–2.1
VSD diameter/aortic root diameter ratio	0.5		

Abbreviations: IVSd, interventricular septum in diastole; IVSs, interventricular septum in systole; LA, left atrium; PA, pulmonary artery; RA, right atrium; RVIDd, right ventricle internal diameter in diastole; RVIDs, right ventricle internal diameter in systole; VSD, ventricular septum defect.

A tentative diagnosis of Tetralogy of Fallot (TOF) or persistent truncus arteriosus (PTA) with ascending aortic aneurysm and rupture of the aorta in the right atrium was made. Thus, considering the poor prognosis for life and especially for athletic use, the owner elected to euthanize the mare.

### 2.5 Post‐mortem findings

**FIGURE 2(a):**
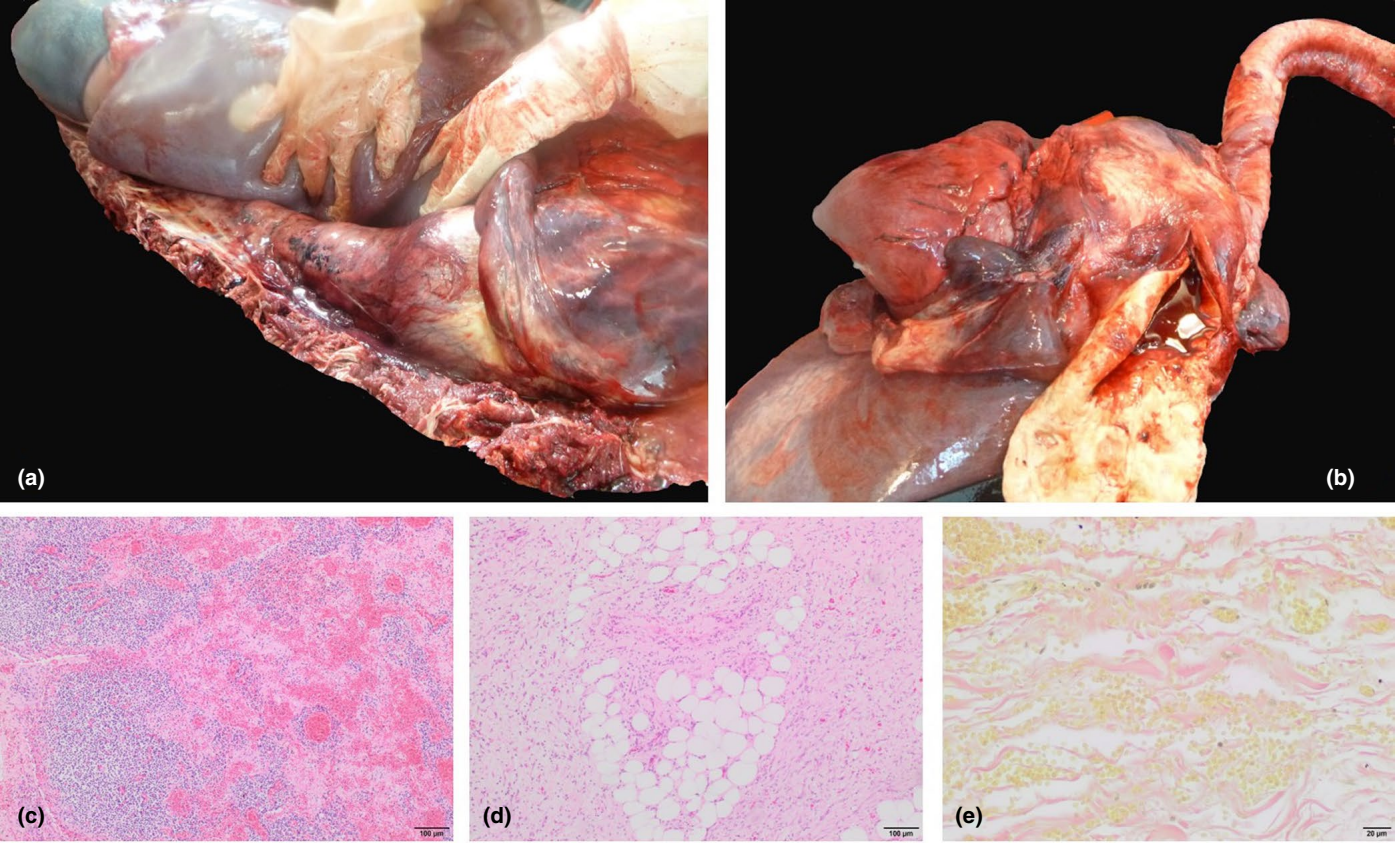
Focally extensive aneurysm extending from the cranial thoracic aorta until the abdominal aorta. (b) The aorta's intima was markedly irregular and thickened. (c) The tunica media are expanded by inflammatory cells, haemorrhage and small amounts of karyorrhectic debris. Haematoxylin and eosin 10x. 2D Tunica media demonstrating adipose tissue infiltrate. Haematoxylin and eosin 10x. 2E. There is multifocal disorganization of the collagen fibres. Van Gieson 40x

The *post‐mortem* examination revealed a 25‐cm‐diameter saccular dilation of the cranial thoracic aorta that extended uniformly until the abdominal aorta (Figure 2a). The aorta's adventitia showed a markedly extensive encapsulated perivascular haematoma. The right atrium was markedly dilated, the right ventricular wall was moderately hypertrophic and there was a 3‐cm‐diameter communication in the interventricular septum (VSD). The aorta communicated with both the right ventricle and right atrium (overriding aorta). The proximal aorta's intima was markedly irregular and thickened diffusely over the distance of 120 cm (Figure 2b). Histologically the tunica media was multifocally expanded by lymphocytes, plasma cells, haemorrhage and a small amount of karyorrhectic debris (Figure 2c). Similar aggregates of inflammatory cells and haemorrhage were moderately to markedly expanding the adjoining adipose tissue (periaortic haematoma). Multifocally, the medial smooth muscle was replaced by moderate amount of adipose tissue (Figure 2d). There was moderate, multifocal disorganization and fragmentation of the elastic lamina, which was more evident by Van Gieson stain (Figure 2e). The vasa vasorum was occasionally cuffed by small numbers of lymphocytes and plasma cells. Multifocally in the intima, reactive endothelial cells were associated with deposits of moderate amounts of fine‐to‐coarse fibrin with enmeshed erythrocytes and few inflammatory cells (fibrin thrombi). Accumulation of mucoid material and disorganized and fragmented laminae in the aortic media completely obliterated *vasa vasorum*.

### 2.6 Diagnosis

Based on the clinical, echocardiographic and *post‐mortem* findings, a final diagnosis was made of an aortic aneurysm with periaortic and intramural haematoma and aortic rupture into the right atrium associated with a tetralogy of Fallot.

## 3 DISCUSSION

This is the first report of a large ascending aortic aneurysm associated with a cardiac congenital malformation in a 5‐year‐old Australian Stock Horse.

Complex or severe CHD usually results in overt clinical signs of cardiac disease in foals within the first days to weeks of life (Gesell & Brandes, 2006; Scansen, 2019). Rarely, horses with CHD present in adult life. Only few reports exist that document complex CHD, such as TOF, in horses older than 1 year (Cargile et al., 1991; Gesell & Brandes, 2006; Taulescu et al., 2016). Interestingly, only one of these cases was older than 5 years and was presented with mild exercise intolerance for most of his life that drastically worsened with increasing age. The two younger horses, a Morgan filly and a Shetland pony mare, presented progressive exercise intolerance, anorexia, weight loss and respiratory distress.

In our case the presence of overriding aorta, VSD, pulmonary stenosis and right ventricular hypertrophy, observed at echocardiography and confirmed at *post‐mortem* examination, is consistent with a diagnosis of TOF. Moreover, the continuity between the aortic and pulmonary valve noted at echocardiography is consistent with a presumptive diagnosis of a type IV PTA, which is considered a severe form of TOF. Four types of PTA have been described: in type I the pulmonary artery is present but arises from the truncal root; in type II the right and left pulmonary arteries originate adjacent to one another from the medial wall of the truncus; in type III the right and left pulmonary artery arise from more widely separated orifices on the medial truncal wall and in type IV the pulmonary blood flow is derived completely from the descending aorta (Scansen, 2019). Based on *post‐mortem* findings of a separation between the aortic and pulmonary artery do not confirm the suspicion of a PTA.

Echocardiography is invaluable in the investigation of all forms of heart disease in the horse (Marr et al., 1998). Nevertheless, necropsy is irreplaceable in the definitive diagnosis and quantification of complex congenital heart defects, as some lesions are challenging to interpret on echocardiography (Kohnken et al., 2018). Indeed, previous reports have documented discrepancies between echocardiographic and *post‐mortem* diagnosis (Hall et al., 2010).

Approximately 74% of human aortic aneurysms occur in the abdominal part and approximately 23% in the thoracic part of the aorta (Baxter, Terrin, & Dalman, 2008). In horses, several cases of aortic root disease have been reported, which are characterized by an aneurysm of the aortic sinus of Valsalva or a tear in the aortic root. This type of aortic rupture usually occurs very close to the junction of the aorta with the heart. Sometimes the aorta ruptures into the right atrium, right ventricle or IVS (Ploeg et al., 2013). A feature of aortic rupture already described by Van der Linde‐Sipman (1985) is the presence of periaortic haemorrhage. These haemorrhages were previously regarded as dissecting aneurysms. However, it has now become clear that these are circumferential cuffs of perivascular haemorrhage formed by leakage of blood out of the ruptured site into the connective tissue surrounding the arteries (Ploeg et al., 2013, 2015). Thus, in this mare, the presence of the periaortic haematoma confirmed the aortic rupture that was found at the level of the right atrium. Considering the multiple and complex congenital malformations and the concomitant presence of aortic rupture, the clinical history without any previous sign of cardiovascular disease is intriguing. Aortic rupture and aortopulmonary fistulation in Friesians can manifest not only as an acute event with haemothorax and death within minutes but also as less acute pathology (Ploeg et al., 2013). In some cases, the aortic tear forms a cuff of perivascular haemorrhage around the aorta and/or the pulmonary artery allowing for stabilization for several weeks (Decloedt, 2019; Ploeg et al., 2013). Accurate *ante‐mortem* diagnosis of this condition is challenging and literature suggests to include aortic rupture in the differential diagnosis when confronted with a Friesian horse with history of recurrent nongastrointestinal–related colic and one or more of the following: ventricular tachycardia, continuous right‐sided murmur, coughing, exercise intolerance, fever, epistaxis, sustained tachycardia and bounding arterial pulse (Decloedt, 2019; Marr et al., 1998; Ploeg et al., 2013; Reef et al., 2014; Taulescu et al., 2016). Although this case was not a breed predisposed to cardiac abnormalities such as Friesians, since admission a nongastrointestinal cause of tachycardia was suspected but few signs of cardiovascular disease were present on physical examination compared with the findings detected at cardiac ultrasound and necropsy. In particular, the absence of an audible murmur was surprising considering the multiple cardiac shunts. Hypothetical explanations include the large size of the VSD, its bidirectional flow and the presence of the aneurysm that could have dampened the murmur. Considering that the reported horse demonstrated a large VSD and a high ratio between the VSD and the aortic root, it was surprising that until recently the horse had lived and exercised well with the congenital defects. Defects likely to result in haemodynamic derangement in horses are those larger than 2.5 cm in a 450–500 kg horse or a ratio of VSD diameter to aortic root diameter greater than 0.4, although other authors speculate that high levels of compensation can be possible (Gesell & Brandes, 2006).

In humans, congenital aneurysm has been linked to connective tissue diseases, such as Marfan's syndrome and Ehlers‐Danlos syndrome (Mork et al., 2019). Recently also mucoid extracellular matrix accumulation (MEMA) has been associated with a primary disorder of collagen and is sometimes observed together with other cardiac defects (Halushka et al., 2016). CHD with or without a collagen alteration in humans predispose to the development of aortic aneurysm (Mork et al., 2019). Accumulation of mucoid material in the aortic media is a finding already described in Friesians and in a Paint mare (Ploeg et al., 2015; Tursi et al., 2018) and has been identified also in this case. Furthermore, in humans alterations of the *vasa vasorum* network play a role in aneurysmal development (Mallat, Tedgui, & Henrion, 2016; Billaud et al., 2018). Obliteration of the vasa‐vasorum, as observed in this case, has been seen in Friesian horses with aortic rupture (Ploeg et al., 2015). Moreover, the congenital malformation and the altered aortic flow together with the periaortic haematoma and the inflammatory infiltrate could have been the cause of the abnormal intimal thickening of the aorta.

**FIGURE 3:**
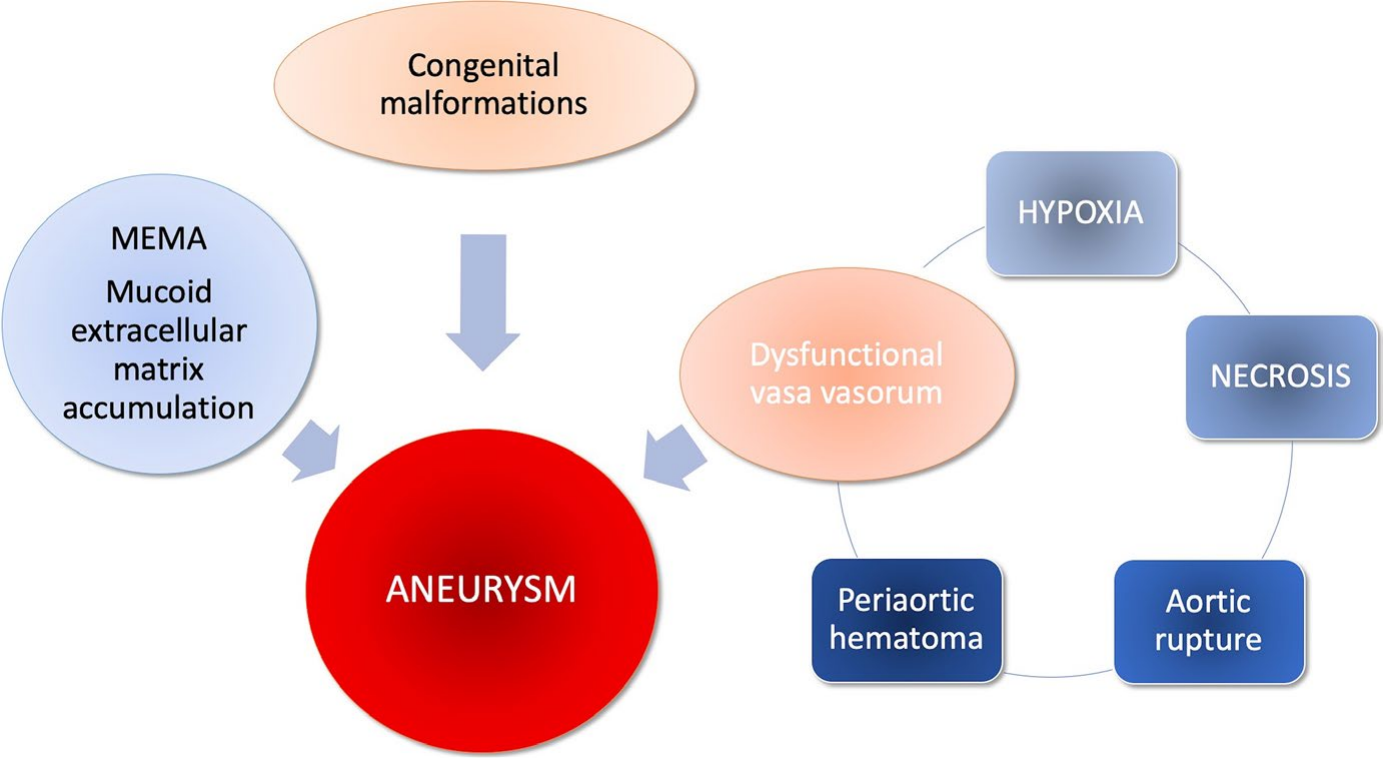
Causes that might have contributed to the development and enlargement of the aortic haematoma in the present case

In summary, in our patient we identified at least four factors that could have contributed at different levels and stages to the development and enlargement of the aortic haematoma: congenital malformation, MEMA and dysfunctional vasa vasorum (Figure 3).

It is difficult to make assumptions regarding the large colon displacement and torsion found at the exploratory celiotomy. A biopsy taken from pelvic flexure during the surgery revealed marked eosinophilic infiltration, a finding typical of reperfusion injury (Rotting, Freeman, Constable, Eurell, & Wallig, 2016), thus, although no macroscopic vascular abnormalities were observed, microscopic changes were present. It is possible that the acute severe pain was related not only to the cardiac disease but also to this acute concomitant gastrointestinal lesion, nevertheless this remains a speculative hypothesis.

## 4 CONCLUSIONS

Although colic is primarily caused by gastrointestinal issues, cardiac disease should be suspected even in absence of murmurs or arrhythmias if unexplained sustained tachycardia is present (Marr et al., 1998). Despite the fact that congenital abnormalities are usually detected in foals, they may sometimes pass unnoticed for several years (Gesell & Brandes, 2006; Scansen, 2019).

## CONFLICT OF INTEREST

None.

## AUTHOR CONTRIBUTION

**Valentina Vitale:** Conceptualization; Investigation; Writing‐original draft. **Gaby Van Galen:** Conceptualization; Investigation; Methodology; Writing‐review & editing. **Malene Laurberg:** Investigation; Writing‐review & editing. **Bridgette Young:** Writing‐review & editing. **Victoria Mciver:** Investigation; Writing‐review & editing. **Marta Wereszka:** Investigation; Writing‐review & editing. **Marina Gimeno:** Conceptualization; Investigation; Methodology; Writing‐original draft; Writing‐review & editing.
